# Appreciating the Strengths and Weaknesses of Transthoracic Echocardiography in Hemodynamic Assessments

**DOI:** 10.1155/2012/894308

**Published:** 2012-02-20

**Authors:** Stephen J. Huang, Anthony S. McLean

**Affiliations:** Department of Critical Care Medicine, Nepean Hospital, Sydney Medical School, Penrith, NSW 2750, Australia

## Abstract

Transthoracic echocardiography (TTE) is becoming the choice of hemodynamic assessment tool in many intensive care units. With an ever increasing number of training programs available worldwide, learning the skills to perform TTE is no longer a limiting factor. Instead, the future emphasis will be shifted to teach the users how to recognize measurement errors and artefacts (internal validity), to realize the limitations of TTE in various applications, and finally how to apply the information to the patient in question (external validity). This paper aims to achieve these objectives in a common area of TTE application—hemodynamic assessments. We explore the strengths and weaknesses of TTE in such assessments in this paper. Various methods of hemodynamic assessments, such as cardiac output measurements, estimation of preload, and assessment of fluid responsiveness, will be discussed.

## 1. Introduction

Hemodynamic assessments form an indispensable part in optimizing fluid status, with the objective of improving adequate tissue perfusion in critically ill patients. In the last decade or two, the practice of critical care medicine is slowly moving away from traditional high-risks invasive procedures wherever possible. Echocardiography, especially transthoracic echocardiography (TTE), has been gaining popularity due to its noninvasiveness where the benefit far outweighs the risk [1]. Its ability to provide vital information about the cardiovascular and hemodynamic status of the patients within a short time frame (within 30 minutes) is another attraction for its use in the critical care setting [2]. A proper focused bedside assessment of cardiac function by TTE can provide answers to important questions about the cardiac function within 10–15 minutes [3]. Assessment for fluid status can also be done within 10–15 minutes. At present, there are no other bedside investigative tools that provide the same level and amount of information as echocardiography. That said, it is important to realize that echocardiography has its strengths and weaknesses. It can suffer from internal and external validity problems. Internal validity refers to the errors associated with the study procedure such as artefacts and measurement errors, and external validity refers to the applicability of the study findings to a particular patients. This article will discuss some of the strengths and validities of TTE in hemodynamic assessments.

## 2. Hemodynamics

**Figure 1:**
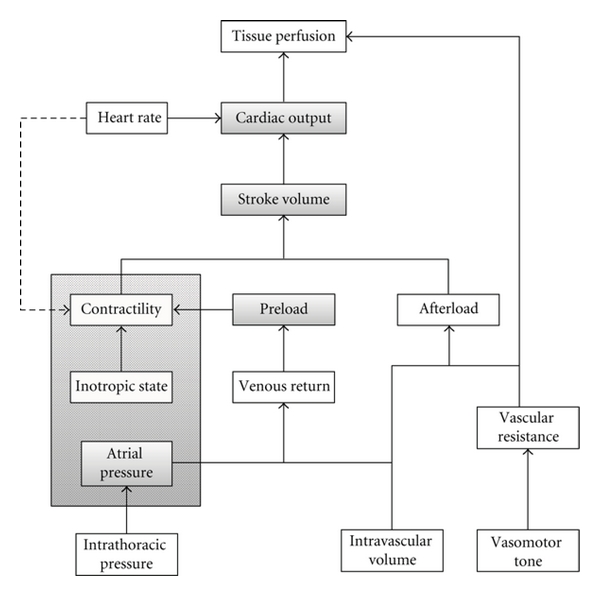
General scheme of hemodynamics.

The term “hemodynamics” is not very well defined but is generally used to refer to “the physics of blood circulation”. It involves the study of the control of circulation and the factors that alter it.  Figure 1 shows the general scheme of hemodynamics in the body. The main function of blood circulation is to ensure adequate tissue perfusion, which is related to two important factors: cardiac output and vascular resistance. Cardiac output is the product of stroke volume and heart rate, where the former can be affected by preload, afterload and cardiac contractility. Afterload, or the tension on myocardial wall during systole, depends on the blood pressure downstream and is determined by the intravascular volume and the resistance of the vasculature where the vasomotor tone is under continuous control by various neurohumoral and local factors. On the other hand, preload, or the tension on the myocardial wall during end-diastole, is determined largely by the venous pressure hence intravascular volume. Cardiac contractility is influenced by (1) the inotropic state, (2) preload by way of the Frank-Starling mechanism, and (3) to a lesser extent, heart rate and rhythm. LV geometry may also affect stroke volume and is best depicted by TTE when compared to blind invasive monitoring system. It is apparent that all these factors are intimately connected.

Left and right heart function aside, the expression “hemodynamics” is often used to denote the assessments of cardiac output, fluid status, and intravascular pressures, the latter often being used as surrogates for afterload (e.g., arterial blood pressure) and preload (e.g., central venous pressure and pulmonary artery occlusion pressure). While vascular resistance can be assessed by various methods, it is based on information derived from other measurements, such as cardiac output and mean arterial blood pressure. It is therefore not a direct measurement and its clinical value is unclear.

## 3. Hemodynamic Assessments by TTE

The use of TTE in hemodynamic assessment is an attractive approach because the procedure is noninvasive and a focused assessment takes less than 20 minutes. However, the biggest drawbacks are (1) it is not a continuous monitoring technique and (2) study quality can be limited by a number of factors including patient's position and habitus, comorbidities, mechanical ventilation, operator expertise, and machine quality. Fortunately, reasonable study quality can usually be obtained in majority of the cases provided that the operator is reasonable skillful such as attained level 1 or basic ultrasound training [3]. Most hemodynamic parameters and other useful information can be extracted even with suboptimal image quality. A standard TTE provides vital information about the heart function (Table 1), the estimation of cardiac output and assessment of preload (fluid status) and fluid responsiveness.

### 3.1. Measurement of Cardiac Output (CO)

Cardiac output is the most often used surrogate for monitoring hemodynamic in intensive care unit (ICU). It is used for guiding treatment especially in patients with shock. TTE can provide a point estimate (“snapshot”) of the CO. CO can be determined by either 2D volumetric methods such as the Simpson's method or Doppler echocardiography. Unfortunately, the 2D image qualities of the critically ill are usually suboptimal hence precluding the use of Simpson's method. Instead, CO can be more reliably determined using Doppler TTE. CO is measured at the left ventricular outflow tract (LVOT), and is based on the mathematical relation of CO = SV × HR, where SV and HR are stroke volume and heart rate, respectively. Echocardiographically, three parameters are needed to work out the CO: (a) LVOT velocity time integral (VTI_LVOT_), (b) LVOT cross-sectional area (CSA), and (c) HR [4]. The VTI is the summation of all velocities per heartbeat and is represented by the area under the curve for each heartbeat. The LVOT velocity is obtained by placing the pulsed-wave Doppler sample gate in the LVOT in apical-5-chamber window. VTI_ LVOT _ is obtained by manually tracing the Doppler velocity spectrum. The process of summation of the velocities is however automated. An average of 3 to 5 consecutive VTI_ LVOT _ is normally used to minimize variability. The CSA of the LVOT is calculated from the diameter of the LVOT obtained from the parasternal long axis window. Measurement of the diameter is done manually, but the calculation of area is automated. Heart rate is obtained by measuring the R-R interval. If accurately done, the TTE obtained CO is comparable to pulmonary artery catheter thermodilution method [5].

**Limitations**

The major limitations of TTE CO measurement are listed in Table 2. One of the major limitations is the lack of continuous tracking ability. Serial measurements can be done, but is laborious and “round-the-clock” availability of sonographers is a problem in many units. Sudden and rapid changes in hemodynamic status mean finding the right operator and setting up the ultrasound machine may be too late in some instances. Interobserver variability may also be an issue.

**Figure 2:**
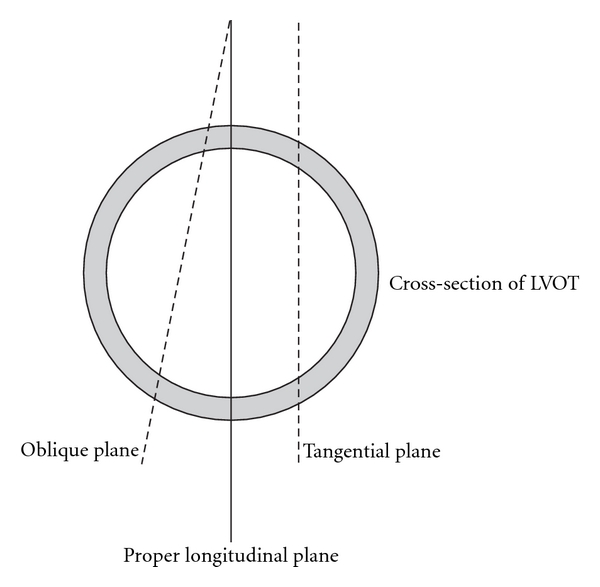
Schematic diagram showing proper and improper ultrasound longitudinal planes in measuring left ventricular outflow tract (LVOT) diameter. Oblique plane is resulted from a tilted angle from the proper plane, while a parallel shift in ultrasound plane results in a tangential planes. Both oblique and tangential planes give rise to underestimation of LVOT diameter. The same is true for measuring inferior vena cava diameter.

Other limitations are related to measurement errors of which there are two: errors in LVOT diameter measurement and in Doppler velocity measurement (Doppler angle error). Measurement of LVOT diameter relies on obtaining a proper longitudinal plane of the LVOT. Slight angulation or lateral misplacement of the transducer will result in obtaining an oblique or tangential plane of the LVOT, hence underestimating the LVOT diameter (Figure 2). Incorrectly identifying the tissue-blood interface may result in under- or overestimation of the diameter. Since the CSA is proportional to the square of the diameter [CSA = *π* × (diameter/2)^2^], any error made will also be squared. A 10% error in diameter will result in approximately 20% error in CSA, hence CO.

Accurate Doppler measurements demand the ultrasound beam being parallel to the blood flow (i.e., Doppler angle = 0°). Deviation from this will result in underestimation of blood flow velocity. While a Doppler angle of 20° results in an acceptable 6% error, a 30° angle will end up with greater than 10% error. It is often forgotten that blood is flowing in a three-dimensional perspective—the 2-dimensional (*X*-*Y*) plane as seen on the screen plus a *Z*-plane which is perpendicular to the screen. Angle correction (for the *X*-*Y* plane) is seldom used in echocardiography as the error in *Z*-plane is unknown. The only remedy is to ensure a proper apical-5-chamber window is used for measurements, and the transducer should be tilted or moved around slightly to obtain the maximal velocity. Foreshortening apical-5-chamber window should be avoided; otherwise CO should not be measured. Of note, motion artefact due to respiration can also lead to angle error by tilting the heart plane up and down. Sometimes, it is difficult to differentiate this motion artefact from the SV variation (see below) in a fluid-depleted but responsive patient. The operator may see alternating 4- and 5-chamber views in concert with the respiratory phase if it is due to motion artefact.

Arrhythmias is another factor that can lead to an measurement error. For patients with atrial fibrillation, the VTI_LVOT_ measurement should be averaged over at least 5 consecutive cardiac cycles. The HR should also be averaged. Ectopic beats should be avoided.

### 3.2. Estimation of Right Atrial Pressure

**Figure 3:**
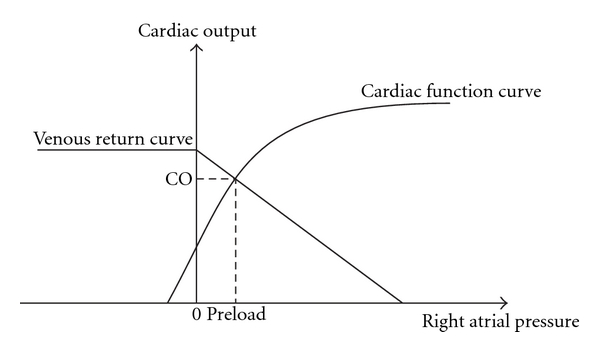
The relationship of venous return and cardiac function in determining cardiac output. As depicted in the venous return curve, venous return reduces with increasing right atrial pressure. The cardiac function curve illustrates the effect of increasing right atrial pressure on cardiac output (CO). Increasing right atrial pressure causes as increase in CO until a plateau (flat portion) is reached. At equilibrium, CO is determined by the point where two curves cross each other. The right atrial pressure at this point is the preload. The cardiac function curve is also known as the Frank-Starling curve.

In the classical Guyton's theory, cardiac output not only depends on the cardiac mechanics, but also on the venous return which determines the right atrial pressure (RAP) which can be approximated by central venous pressure (Figure 3). The inferior vena cava (IVC) has long been used to predict RAP in nonventilated patients [6]. In response to a change in intrathoracic pressure during respiration the IVC diameter changes. The diameter is the largest during expiration, and is the smallest during inspiration or sniffing. The IVC collapsibility index, defined as the difference in IVC diameters (during expiration and inspiration) divided by the maximal diameter (or (*D*_expiration_ − *D*_inspiration_)/*D*_expiration_ where *D* is the diameter), has been shown to be correlated to RAP [6, 7]. Various cutoffs have been used to predict RAP [6, 8].

The IVC collapsibility index is obtained from the subcostal window. The patients are asked to perform a brief rapid inspiration or a sniff. The maximum diameter of the IVC is obtained during expiration, and minimum diameter is obtained during an inspiratory or a sniff maneuver.

**Limitations**

Contrary to traditional belief, RAP and central venous pressure have been proven to be poor predictors for fluid status (or blood volume) in critically ill patients [9, 10]. Since IVC diameter and collapsibility index are used as surrogates to estimate RAP, it follows that such information will not be very useful in predicting fluid status in critically ill patients despite the fact that IVC collapsibility index correlates with RAP.

The main limitation of the applicability of IVC collapsibility index is its applicability in mechanically ventilated patients. Studies that demonstrated good correlations between IVC collapsibility index and RAP were mostly performed in spontaneously breathing patients [6, 7]. The correlations in mechanical ventilated patients were poor [11, 12]. Compounded to the mechanical ventilation effects are a number of confounding factors that also affect the IVC diameter and collapsibility in critically ill patients. For example, right heart failure, significant tricuspid regurgitation, and supine body position, which are common in critically ill patients, are known to dilate the IVC [12–15]. Despite these, an IVC diameter of ≤12 mm has 100% specificity, with only 25% sensitivity, of predicting an RAP of 10 mmHg or less in mechanically ventilated patients [12].

The main technical limitation results from motion artefact are due to diaphragmatic and abdominal wall movements. IVC is commonly displaced inferiorly by the diaphragm during inspiration or sniffing, and affects the measurements especially when M-mode is used. Abdominal wall motion during sniffing can displace the transducer, hence the ultrasound plane, during the measurements. Care must therefore be taken to minimize such displacements while maintaining the ability to capture the changes in diameter during inspiration or sniffing. Measurements are best done in 2D mode with high frame rate to minimize such measurement errors.

## 4. Determination of Fluid Responsiveness

**Figure 4:**
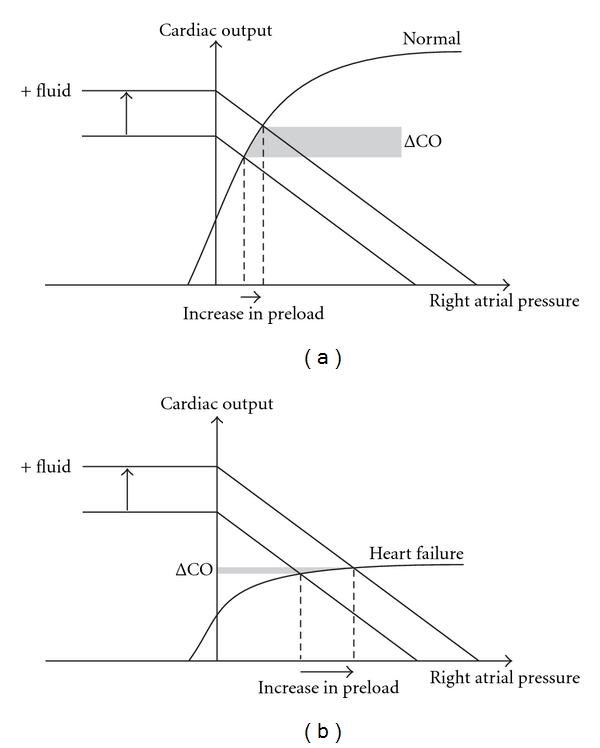
The effect of fluid administration on cardiac output. Fluid administration results in an upward shift in venous return curve. As a result, there is an increase in preload hence cardiac output (ΔCO) (a). Note that the crossing points between the two curves are on the steep part of the cardiac function (Frank-Starling) curve. In heart failure, the Frank-Starling curve is lowered, and the crossing point is at the flat portion of the curve (b). Fluid administration, although it increases the preload, does not result in an increase in CO in the latter case.

It is a well-known fact that administering fluid to patients whose hearts are operating at the flat (top) portion of the Frank-Starling (preload versus SV) curve is harmful to patients [16]. Various techniques have been developed to identify those “fluid responsive” patients who will benefit from fluid administration, that is, those whose ventricles are operating at the steep (ascending) part of the Frank-Starling curve (Figure 4). In a systematic review and meta-analysis of clinical study, it was found that approximately half (52.9%) of the study population were responders [17]. Two approaches have been adopted to predict fluid responsiveness: (a) volume expansion and (b) respiratory variation in SV or its surrogates.

### 4.1. Volume Expansion

The volume expansion approach utilizes the fact that if preload is increased acutely in those who will benefit from fluid administration, an accompanying increase in SV (or CO) should be observed (Figure 4) [18]. Volume expansion can be achieved via (a) passive leg raising (PLR) or (b) volume challenge—rapid infusion of a fixed volume (e.g., 500 mL) of fluid within 15–30 min [19]. In response to acute volume expansion, an increase in SV or its surrogates, such as echocardiographic LVOT or aortic flow VTI (and CO), are expected. In patients whose ventricles are operating at the flat portion of the Frank-Starling curve, the change in stoke volume is less apparent (Figure 4). Studies involving the use of PLR reported that an increase in CO by 12–15% offers high specificities (>90%) and sensitivities (>80%) in discriminating fluid responder from nonresponder [17]. PLR-induced increase in SV or CO displays a good correlation with volume challenge [17, 19]. While SV and CO are reported, their surrogates such as VTI_LVOT_ or aortic flow VTI (VTI_aortic_) are normally used. VTI_aortic_ can be measured either in the apical-5-chamber window or the suprasternal window using continuous wave Doppler.

### 4.2. Respiratory Variation

**Figure 5:**
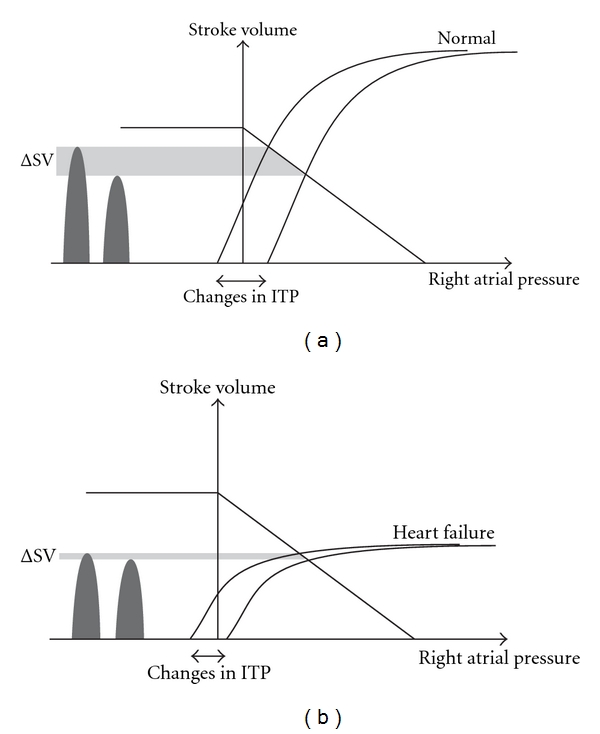
The effect of intrathoracic pressure on cardiac output. Both spontaneous breathing and mechanical ventilation result in a cyclical change in intrathoracic pressure (ITP). Depending on the mode of ventilation, the change in ITP is accompanied by a cyclical shift in the Frank-Starling (cardiac function) curve which results in a cyclical change in preload. If the crossing point is on the steep part of the curve, shifting of the curve would result in a change in stroke volume (SV) (or cardiac output). This respiratory induced cyclical change in SV is known as SV variation (a). In heart failure, such change in SV may be less apparent because the crossing point is at the flat portion of the curve (b).

During steady respiratory effort, changes in intrathoracic pressure alters the heart-lung interaction mechanics leading to a regular fluctuation of SV [20]. In mechanically ventilated patients, the LV SV is at its greatest at the end of the inflation period. This is due to an increase in pleural pressure and LV preload. During expiration, the reduction in LV preload decreases the SV. This respiratory variation in SV (SV variation or SVV) is exaggerated in fluid responders whose ventricles are operating at the steep part of the Frank-Starling curve (Figure 5). For those (non-responders) whose ventricles are operating at the flat portion of the curve, the SVV is blunted (Figure 5) [19]. SVV is defined as (SV_max_ − SV_min_)*⁄*SV_mean_ × 100%, where the subscripts max, min, and mean stand for maximum, minimum, and average, respectively. In TTE, the percentage variation in VTI_LVOT_ or VTI_aortic_ is normally used as a surrogates for SVV [21, 22].

Respiratory variation in right atrial pressure has also been used to predict fluid responsiveness [23, 24]. As a surrogate for RAP, the changes in IVC diameter with respiratory variation has also been shown to be a satisfactory predictor for fluid responsiveness in mechanically ventilated patients. Barbier et al. demonstrated a percentage distensibility of IVC, defined as (IVC_max_ − IVC_min_)*⁄*IVC_min_, of 18% offers 90% sensitivity and specificity in discriminating fluid responders from non-responders [25]. On the other hand, Feissel et al. found that a percentage variation in IVC, defined as (IVC_max_ − IVC_min_)*⁄*IVC_mean_, of 12% can predict fluid responder with >90% sensitivity and specificity [26]. Of note, IVC distensibility and variability should not be confused with IVC collapsibility (see above). While the former two predict fluid responsiveness, the latter predicts RAP. The use of superior vena cava (SVC) collapsibility index has also been used, but transesophageal echocardiography is necessary to visualize the SVC [27].

### 4.3. Limitations 

One of the main concerns of using respiratory variation is its applicability in spontaneously breathing patients or in those not in mandatory controlled mode [28]. In one study, SVV was found to be a poor predictor for fluid responder in patients with septic shock on pressure support mode [29]. Breath-by-breath changes in inspiratory effort may alter the intrathoracic pressure hence the regular SVV or IVC variations required for such analysis. Further, variations in tidal volume and duration of respiratory cycle in noncontrolled mode may negate the use of SVV or IVC variations. Charron et al., however, showed that VTI_aortic_ was still a satisfactory predictor for fluid responsiveness even when the tidal volume was increased [30]. However, no follow-up study was done in this regard.

Elevated intraabdominal pressure is another confounding factor for assessment of fluid responsiveness. Intra-abdominal hypertension has been shown to render the use of PLR useless in predicting fluid responsiveness [31]. Animal studies also demonstrated that the normal cutoffs for SVV are not helpful in predicting fluid responsiveness [32, 33]. Increase in respiratory rate in mechanically ventilated patients reduces the VTI_aortic_ variations rendering the use of SVV useless [34]. Intra-abdominal hypertension can also affect the size of IVC and its response to respiration.

## 5. Conclusion

**Figure 6:**
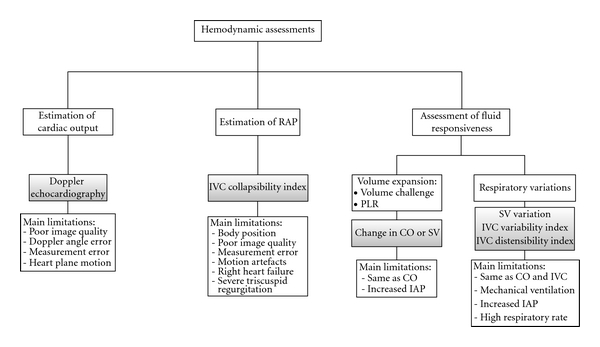
Summary of hemodynamic assessment by transthoracic echocardiography.

TTE has proven itself to be an indispensible critical care tool in recent years. Although its main uses are for exploring the cardiac function, its applications in hemodynamic assessment are increasingly popular. Estimations of cardiac output and right atrial pressure, and ascertaining the fluid status of the patients, are common uses of TTE (Figure 6).

Hemodynamic assessment by TTE is confronted by two major limitations: internal and external validities. Internal validity stems mainly from technical limitations including image quality and measurement errors. Some of these can be minimized by quality control and skill improvement over time. External validity concerns with the applicability and appropriateness of using a particular TTE measurement to answer a specific hemodynamic question. In face of the issue of external validity, the operator should constantly ask himself or herself whether a particular TTE measurement is valid (suitable) for the patient in question. The power of echocardiography can only be unleashed through the understanding of its strength and limitations more fully.

## Figures and Tables

**Table 1:** Common cardiac information which can be provided by a standard TTE.

Left heart	(i) Dimensions: chamber sizes and thickness
	(ii) Left ventricular ejection fraction
	(iii) Regional wall motion abnormalities
Right heart	(i) Dimensions: chamber size and thickness
	(ii) Right ventricular systolic function: FAC or TAPSE
	(iii) Signs of pressure or volume overload
Valvular pathologies	(i) Regurgitations
	(ii) Stenoses
	(iii) Prolapses
	(iv) Presence of vegetation
Aorta	(i) Dilatation
	(ii) Dissection
Estimation of pressures	(i) Pulmonary artery systolic pressure
	(ii) Left ventricular filling pressure
	(iii) Transvalvular pressure gradients
Other	Pericardial effusion and tamponade

FAC: fractional area contraction; TAPSE: tricuspid annular plane systolic excursion.

**Table 2:** Limitations of cardiac output measurements in ICU by TTE.

Cannot provide continuous monitoring
Measurements and accuracies can be affected by:
(i) Patient's position
(ii) Patient's condition: for example, lung hyperinflation,
cutaneous emphysema, trauma, wound
(iii) Effects of mechanical ventilation
(iv) Suboptimal ultrasound windows: poor image quality
(v) Heart plane motion during measurements
(vi) Doppler angle error: poor angle alignment
(vii) Arrhythmias
